# Mechanics of cell-cell junctions

**DOI:** 10.1016/j.bpj.2023.07.011

**Published:** 2023-07-20

**Authors:** Yufei Wu, Sean X. Sun

**Affiliations:** 1 Department of Mechanical Engineering, Johns Hopkins University, Baltimore, Maryland; 2 Institute for NanoBioTechnology, Johns Hopkins University, Baltimore, Maryland; 3 Center for Cell Dynamics, Johns Hopkins School of Medicine, Baltimore, Maryland

## Abstract

Tissue cells in epithelial or endothelial monolayers are connected through cell-cell junctions, which are stabilized by transmembrane E-cadherin bonds and intracellular actin filaments. These bonds and junctions play a crucial role in maintaining the barrier function of epithelia and endothelia and are believed to transmit forces between cells. Additionally, E-cadherin bonds can impact the shape of cell-cell junctions. In this study, we develop a continuum mechanical model of the cell-cell junction by explicitly incorporating the cell membrane, distributions of E-cadherin bonds, cytoplasmic fluid pressure, and F-actin dynamics. The static force-balanced version of the model is able to analyze the influences of cell cortical tension, actin dynamics, and cytoplasmic pressure on the junction shape and E-cadherin bonds. Furthermore, an extended model that incorporates fluid flow, across the cell boundary as well as around the cell, is also examined. This model can couple cell-shape changes with cell cortical tension and fluid flow, and predicts the additional effect of fluid motion on cell-cell junction mechanics. Taken together, our models serve as an intermediate link between molecular-scale models of cell-junction molecules and cell-scale models of tissue and epithelia.

## Significance

We develop a molecular model of the cell-cell junction that can predict the shape and forces within the junction. The model considers several key elements, including the mechanics of E-cadherin bonds, membrane and cortical tension, actin dynamics, cytoplasmic pressure, and fluid flow within and between cells. The model can be extended and generalized to understand the mechanical behavior of multicellular epithelial tissues.

## Introduction

The adherens junction is an important structure in mediating cell-cell adhesion and multicellular signal transduction (1,2,3). In epithelial tissues, the adherens junction is responsible for establishing connections between cells through transmembrane E-cadherin bonds. These bonds span the neighboring plasma membranes and, on the cytoplasmic side, interact with numerous cytoplasmic proteins. Cell-cell junctions are also locations where other membrane-spanning complexes are found: for example, gap junctions are pores directly connecting the cytoplasms of adjacent cells (4). Other related junction structures are desmosomes (5) and tight junctions (6), which involve additional molecules that further stabilize and seal the cell-cell contact region.

**Figure 1:**
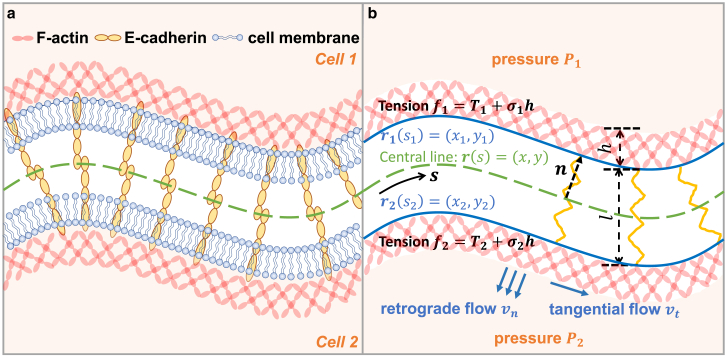
(*a*) Schematic illustration of an adherens junction. The junction contains two cell membranes and E-cadherin bonds which mediate cell adhesion. Attached to E-cadherin is the F-actin network, which helps reinforce the junctional stability. In the actin network, there is both normal (retrograde) and tangential flow. (*b*) Illustrations of kinematic variables considered in the model. The main factors in the mechanical model include cytoplasmic hydrostatic pressures $P_{1},P_{2}$, membrane tensions $T_{1},T_{2}$, and cortical tensions $\sigma_{1},\sigma_{2}$. E-cadherin bonds are modeled as linear springs. The retrograde and tangential actin flow causes frictional forces in the normal and tangential direction, respectively. The junction is represented by the central line (r), which is the mean curve of two membranes ($r_{1}$ and $r_{2}$). To see this figure in color, go online.

Some of the major proteins in the cytoplasmic side of cell-cell junctions are *α*- and *β*-catenins, p120, and F-actin (7,8). In a stabilized adherens junction, E-cadherins are indirectly connected to the F-actin network via these proteins (9). Since E-cadherin bond formation is generally favorable because of bond formation free energy, cell-cell junctional bonds have been proposed to be involved in cell sorting in an epithelium (10). Physical epithelial models such as the vertex model (11,12,13,14) and cellular Potts models (15,16) have been used to describe cell-cell junction shape and epithelial morphology using a conserved free-energy-driven approach. These models typically treat cell-cell junction adhesion using cell-surface energy. However, since E-cadherin can be attached to the dynamic F-actin network, the attachment of E-cadherin to actin filaments can involve additional nonequilibrium effects (Fig. 1 *a*) (17,18). For example, F-actin interacts with force-producing molecules such as myosin, and F-actin undergoes rapid turnover via polymerization and depolymerization; therefore, the cell-cell junction is not a static structure. Rather, it is a dynamic system where energy dissipation is necessary to maintain cell-cell adhesion and the junction structure (19). Therefore, cell-cell junctions are active mechanical structures. The standard vertex models with homogeneous junction properties cannot capture any spatial variations in junction tension and deformation (e.g., asymmetric adherens junction contraction (20)). While there are modifications of vertex models that incorporate such mechanical heterogeneity (21), these models still neglect the shape of the junction, treating it as a straight line. The junctional shape also potentially contains information about the physical microenvironment around the cell (e.g., fluid pressure, extracellular matrix stiffness). When hydraulic pressure and tension are unevenly distributed along the cell-cell junction, the junctional shape becomes wavy instead of straight (22,23). Some models rely on the Young-Laplace law to relate the pressure difference across a junction to the tension in the junction (24,25), allowing for consideration of the junction shape and potential spatial variation in pressure and tension. However, these models do not take into account E-cadherin bonds and the F-actin dynamics, which are crucial components in cell-cell junctions. An active mechanical model of cell-cell junction that explicitly describes E-cadherin bond force, membrane tension, F-actin flow, and other potential mechanical variables would further advance our understanding of epithelial mechanics and multicellular movement.

In this paper, we mechanically model the cell-cell junction by incorporating membrane tension, cortical tension, E-cadherin bond mechanics, cytoplasmic pressure, and E-cadherin interactions with F-actin and cortical flow. We also consider the influence of fluid flow inside and outside of the cell and pressure/osmolarity-driven flow across the cell-cell boundary through the permeable membrane and gap junctions. These elements are combined to obtain cell-cell junction shape and E-cadherin bond stress. We examine several versions of this model: a static E-cadherin mechanical equilibrium limit that includes F-actin retrograde flow, a dynamical version that includes E-cadherin movement in the membrane and tangential actin flow, and finally a fully dynamical version where the cell boundary movement is coupled to Stokes fluid flow together with the permeable cell membrane. Results show that cell-cell junction shape and E-cadherin distribution and mechanical stretch are influenced not only by cell cortical tension and cytoplasmic pressure but also by actin dynamics. Moreover, using an immersed boundary Stokeslet approach, we show that fluid flow through the cell-cell junction can impact junction shape and the overall movement of cells that are adhered together. This approach can be extended to multicellular clusters and epithelial tissues, although actin and fluid flows should be treated on a consistent and equal footing. The fully dynamical model can be considered a consistent approach to compute multicellular mechanics and movement in general.

### Static mechanical model of adherens junction with actin retrograde flow

Fig. 1*b* shows an illustration of the cell-cell junction and kinematic variables considered in the model. Here we model the E-cadherin bonds (which contain a pair of E-cadherin molecules) between two cells as linear springs, the force of which is proportional to the strain: $F_{cad}=k\varepsilon$, where $\varepsilon =\left(l-l_{0}\right)/l_{0}$. $l_{0}$ is the rest length of the cadherin bond. The stiffness *k* is proportional to the density of E-cadherin and could be spatially varying. On average, E-cadherin bonds should be perpendicular to each cell surface. Therefore, the bond force is in the normal direction (26). In addition to the force of E-cadherin bonds, the hydrostatic pressure *P* and cell-surface tension *f* are also involved in the force balance. The cell-surface tension contains both the cell-membrane tension (*T*) and cortical tension (*σ*): f=T+σh, where *h* is the thickness of cortex. Furthermore, the cortical tension is composed of an active part $\sigma_{a}$ and a passive part $\sigma_{p}$. Generally speaking, the active stress of actin network is much larger than the passive stress and membrane tension. Therefore we can approximate the combined surface tension by the active stress $\sigma_{a}$ only, which is assumed to be proportional to the F-actin concentration $\theta_{n}$. This assumption can be relaxed without difficulty, but additional equations need to be introduced that make the model more complex.

During actin retrograde flow, actin filaments slide slowly relative to E-cadherin, and the junctional proteins form transient attachments between E-cadherin and F-actin. The formation and detachment of these transient bonds generate a frictional force (27). This frictional force is in the same direction as retrograde flow (perpendicular to the cell membrane) and proportional to the velocity of actin flow: $F_{f}=\mu_{f}v_{n}$ (27), where $\mu_{f}$ is related to microscopic kinetics of the E-cadherin-actin bond. In this paper, all model variables are in two dimensions.

Taking into account all the forces and the assumption that E-cadherins are anchored into the cell membrane, we can write down the force balance in the normal direction for each cell surface (23,28):

(1) $$P_{1}-f_{1}H_{1}+k\varepsilon -\mu_{f}v_{nn,1}=0\text{,}$$

(2) $$P_{2}+f_{2}H_{2}+k\varepsilon -\mu_{f}v_{nn,2}=0\text{,}$$

where subscripts 1 and 2 denote the upper and lower cell, respectively. $H_{i}$ is the mean curvature of each cell surface. $v_{nn,i}$ is the actin velocity component normal to the cell surface (i=1,2). The direction pointing toward the interior of the cell is defined as positive. The force terms $F_{cad}=k\varepsilon$ and $F_{f}=\mu_{f}v_{n}$ both have the unit of force per unit area (kPa), which is consistent with the unit of hydrostatic pressure $P_{1},P_{2}$.

In the force balance (1), (2), we have not included contributions from membrane and cortex bending. For the membrane, using the Helfrich theory, the relevant length scale where bending is important is ≈$\sqrt{\kappa /2\gamma }$ (29), where *κ* is he bending modulus and *γ* is the membrane tension. Using experimental estimates, *κ* is about 20 $k_{B}$ T and *γ* is very low, about 0.005 $k_{B}$ T/nm^2^. This gives the length as approximately 50 nm. Therefore, with length scales less than 50 nm, bending is important. For length scales larger than 50 nm, the membrane is also likely to be wrinkled due to thermal fluctuations. Here, the junctions are micrometers in length, and therefore we neglect bending contributions. For the cytoskeletal cortex, because F-actin undergoes cycles of polymerization/depolymerization, the cortex turns over rapidly on the timescales of 10 s. Therefore, for timescale less than 10 s, we expect bending contributions, but for longer timescales, any residual stress in bending would be removed after turnover. We note that there might be other types of cytoskeleton, e.g., spectrin and intermediate filaments, which may turnover at a different rate. In such a case where there are different timescales of turnover, bending of the cortex should be included in the discussion.

Let $r_{1}\left(s_{1}\right)$ and $r_{2}\left(s_{2}\right)$ be the centerline through the upper and lower cell membranes, respectively. $s_{i}\left(i=1,2\right)$ is the arclength of the upper (lower) cell membrane. The cell-cell boundary or the midpoint r(s) is the average of two membrane centerlines: $r=\left(r_{1}+r_{2}\right)/2$. With the assumption that E-cadherin bonds are perpendicular to the central line (26) as shown in Fig. 1 *b*, the individual cell-membrane shapes can be written as

(3) $$r_{1}\left(s\right)=r\left(s\right)+\frac{l}{2}n\left(s\right)\text{,}$$

(4) $$r_{2}\left(s\right)=r\left(s\right)-\frac{l}{2}n\left(s\right)\text{,}$$

where all variables are functions of *s*, the arclength along the central line r. n is the vector perpendicular to the central line (Fig. 1 *b*). Furthermore, we can write the E-cadherin length *l* as $l\left(s\right)=\left|r_{1}\left(s\right)-r_{2}\left(s\right)\right|$. Because the length of the E-cadherin bond is very small when compared to the radius of curvature of the central line, we naturally have the condition Hl≪1, where *H* is the curvature of the central line. We also assume E-cadherin length varies slowly along the adherens junction, which implies dl/ds≪1.

Let $r_{1}=\left(x_{1},y_{1}\right),r_{2}=\left(x_{2},y_{2}\right)$; the curvatures can then be expressed as $H_{i}\left(s_{i}\right)=x_{i}^{\prime }\left(s_{i}\right)y_{i}^{\prime \prime }\left(s_{i}\right)-x_{i}^{\prime \prime }\left(s_{i}\right)y_{i}^{\prime }\left(s_{i}\right);\left(i=1,2\right)$, where the derivatives are with respect to $s_{1},s_{2}$, respectively. With the assumption that Hl≪1 and dl/ds≪1, we obtain the following approximation: $ds_{i}/ds=\sqrt{{\left(1+Hl\right)}^{2}+l^{\prime 2}}\approx 1\left(i=1,2\right)$. The equation of normal force balance (Eqs. 1 and 2) can then be expressed as

(5) $$P_{1}-\frac{f_{1}y_{1}^{\prime \prime }}{\sqrt{1-y_{1}^{\prime 2}}}+\frac{k}{l_{0}}\left(\sqrt{{\left(x_{1}-x_{2}\right)}^{2}+{\left(y_{1}-y_{2}\right)}^{2}}-l_{0}\right)-\mu_{f}v_{nn,1}=0\text{,}$$

(6) $$P_{2}+\frac{f_{2}y_{2}^{\prime \prime }}{\sqrt{1-y_{2}^{\prime 2}}}+\frac{k}{l_{0}}\left(\sqrt{{\left(x_{1}-x_{2}\right)}^{2}+{\left(y_{1}-y_{2}\right)}^{2}}-l_{0}\right)-\mu_{f}v_{nn,2}=0\text{,}$$

(7) $$x_{1}^{\prime }=\sqrt{1-{y^{\prime }}_{1}^{2},}$$

(8) $$x_{2}^{\prime }=\sqrt{1-{y^{\prime }}_{2}^{2}.}$$

All the derivatives are with respect to central arc length *s*, and we assume that there is a 1-to-1 correspondence between $y_{i}$ and $x_{i}$. For boundary conditions, we assume that the E-cadherin length is fixed at both ends of the junction. Consequently, we have $y_{1}\left(0\right)=l_{b1},y_{1}\left(s_{0}\right)=l_{b2},y_{2}\left(0\right)=y_{2}\left(s_{0}\right)=0,x_{1}\left(0\right)=0,x_{2}\left(0\right)=0$, where $s_{0}$ is the total length of junction. Other types of boundary conditions can be considered also. Nondimensionalizing the force balance equations, we obtain two important dimensionless parameters: pressure $\tilde{P}=P/k$ and tension $\tilde{f}=fl_{0}/\left(ks_{0}^{2}\right)$.

In this work, for simplicity, we assume that the actin network in the cortex is incompressible. Therefore the retrograde flow velocities satisfy $\nabla \cdot \left(\theta_{n}v_{n}\right)=0$, where $v_{n}$ is the velocity vector of actin network and $\theta_{n}$ is the F-actin concentration. In this case, the normal component of the actin velocity, $v_{nn}$, can be further simplified as $\theta_{n}v_{nn}=J_{n}$, with the approximation that the streamlines of actin flow are parallel to each other and tangential action flow is negligible. $J_{n}$ is the actin polymerization rate. Typically, actin filaments are polymerized at the outer boundary of the cell cortex and depolymerized at the inner boundary, thus generating retrograde flow toward the cell center. Coupled with the assumption that the cortical tension (*σ*) is proportional to F-actin concentration ($\theta_{n}$), the shape equation (Eqs. 5, 6, 7, and 8) can be numerically solved just by prescribing the actin polymerization rate together with pressure and cortical tension.

#### Junction shapes and E-cadherin length distributions

**Figure 2:**
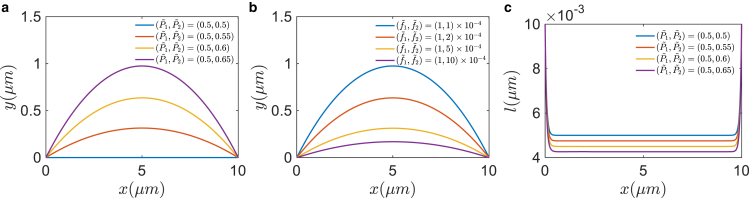
Junction shape and E-cadherin length distribution in the absence of retrograde flow. The axis origin is placed at the left end of the centerline r(s). The *x* axis represents the direction connecting the two ends of the centerline, while the *y* axis is perpendicular to the *x* axis. (*a*) Effects of dimensionless pressure on junction shape. (*b*) Effects of dimensionless tension on junction shape. With fixed pressure difference, larger tension causes smaller curvature. (*c*) E-cadherin bond length along the junction. The bond length, l(s), is almost compressed everywhere in the junctional region. In(*a*) and (*c*), only the pressure is varied while tension is fixed as $\tilde{f_{1}}=\tilde{f_{2}}=10^{-4}$; in (*b*), only tension is varied while pressure is fixed as $\tilde{P_{1}}=0.5,\tilde{P_{2}}=0.65$. In all the results above, the boundary condition for the E-cadherin bond length is set as $l_{b1}=l_{b2}=l_{0}=0.01$*μ*m. To see this figure in color, go online.

We first discuss cell-cell junctional shape without actin flow, i.e., we neglect the frictional force term in Eqs. 1 and 2. Fig. 2 shows different junction shapes (represented by the central line of two membranes) and length distributions of E-cadherin bonds under different conditions. In the calculation, we set the horizontal length of the junction to be $x_{0}=10\;\mu$m and the lengths of the E-cadherin bond at both ends to be the same as rest length: $l_{b1}=l_{b2}=l_{0}=0.01$ *μ*m. The E-cadherin stiffness is a constant: k=1.5 kPa (30,31,32). As expected, the structure of the junction is mainly determined by two dimensionless parameters: pressure $\tilde{P}$ and tension $\tilde{f}$. When fixing the tensions as $\tilde{f_{1}}=\tilde{f_{2}}=10^{-4}$ and increasing $\Delta \tilde{P}$, the junction bends to the low-pressure side and the curvature increases (Fig. 2 *a*). When fixing the pressure as $\tilde{P_{1}}=0.5,\tilde{P_{2}}=0.65$, the junction curvature decreases with the increase of tension $\tilde{f_{2}}$ (Fig. 2 *b*). It is worth noting that in absence of forces from the actin network, E-cadherin bonds are compressed almost everywhere in the junctional region due to the hydrostatic pressure from the two cells. The average length decreases with increasing pressure (Fig. 2 *c*).

**Figure 3:**
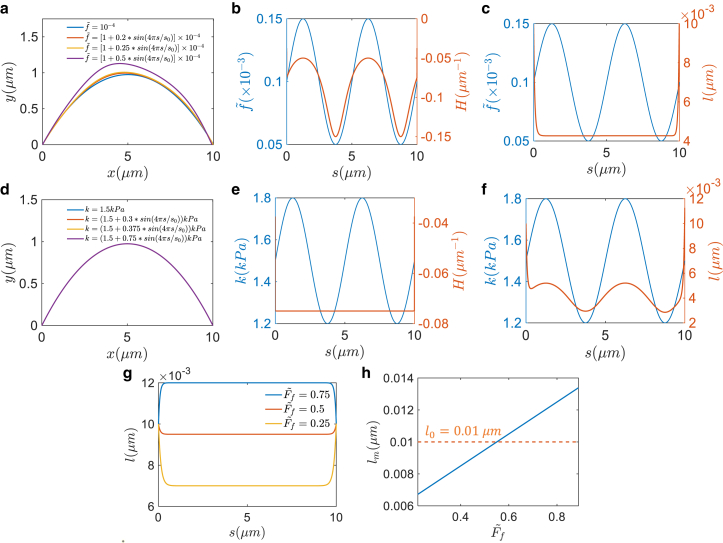
Effects of spatially varying tension and E-cadherin bond stiffness on the junction configuration. (*a*–*c*) Junction shape, curvature, and E-cadherin bond length when tension $\tilde{f}$ is varied spatially (*blue line*). Curvature is trivially correlated with tension, but E-cadherin bond length is little affected by tension. In the calculation, only tension is varying while pressure is fixed as $\left(\tilde{P_{1}},\tilde{P_{2}}\right)=\left(0.5,0.6\right)$. In (*b*) and (*c*), $\tilde{f}=\left[1+0.5\;\sin\left(4\pi s/s_{0}\right)\right]\times 10^{-4}$. (*d*–*f*) Same results with spatially varying E-cadherin stiffness. E-cadherin length instead of junction curvature is more influenced by bond stiffness. In this calculation, only stiffness is varied while pressure and tension are fixed as $\left(\tilde{P_{1}},\tilde{P_{2}}\right)=\left(0.5,0.6\right),\tilde{f_{1}}=\tilde{f_{2}}=10^{-4}$. In (*e*) and (*f*), $k=\left[1.5+0.3\;\sin\left(4\pi s/s_{0}\right)\right]kPa$. (*g*) E-cadherin bond length with varying retrograde flow velocity. Both tension and pressure are fixed. With large enough retrograde flow, the E-cadherin bond can change from a compressed to an extended state. (*h*) Effect of retrograde flow velocity on the E-cadherin bond length at the middle point of cell-cell junction. In all the results above, the boundary condition for the E-cadherin bond length is set as $l_{b1}=l_{b2}=l_{0}=0.01$*μ*m. To see this figure in color, go online.

Along the cell-cell junction, there could be spatial variations in tension *f* and E-cadherin stiffness *k* due to different distributions of F-actin and E-cadherin. Our model can predict their effects on the junction shape. Fig. 3, *a*–*c* show the influence of spatial variation of tension on shape and E-cadherin length. Two cell tensions are set the same and take the form of a sine function. In general, the shape shows a nonuniform pattern corresponding to the tension variation. The curvature is positively correlated with tension. Since the curvature is negative, the absolute value should be negatively correlated with tension, which means larger tension corresponds to a flatter junction. However, the tension variation has little influence on the E-cadherin length, as shown in Fig. 3 *c*. Different from the tension variation case, when spatially varying the stiffness of E-cadherin, the junction shape is little changed but the E-cadherin length is affected (Fig. 3, *d–f*). Larger stiffness corresponds to longer bonds, which means that stiffer E-cadherin bonds are more difficult to compress. In all the calculations, the pressure is set as $\left(\tilde{P_{1}},\tilde{P_{2}}\right)=\left(0.5,0.65\right)$. The stiffness and tension are set as k=1.5 kPa and $\tilde{f_{1}}=\tilde{f_{2}}=10^{-4}$ when they are fixed.

The presence of F-actin retrograde flow can change the state of E-cadherin from compression to stretching when the flow velocity is increased (Fig. 3 *g*). The E-cadherin length increases linearly with the frictional force from the retrograde flow (Fig. 3 *h*). Here the frictional force is normalized by the E-cadherin stiffness: $\tilde{F_{f}}=F_{f}/k$. In the calculation, the pressure is set as $\left(\tilde{P_{1}},\tilde{P_{2}}\right)=\left(0.5,0.6\right)$ and we assume $\tilde{f_{1}}=\tilde{f_{2}}$. The polymerization rate is set as constant: $J_{1}=J_{2}=1$ (s^−1^*μ*m^−2^), frictional coefficient $\mu_{f}=1$ (kPa · s/*μ*m). $f=\zeta \theta_{n}$, where ζ=1 (kPa ·μm^4^). In a more complex model, forces from E-cadherin will also impact actin retrograde flow and possibly myosin contraction. Previous work has shown that the actomyosin network has mechanosensing properties (33,34). Therefore, there is a natural mechanosensing system at the cell-cell junction that can sense the E-cadherin adhesions between cells.

It is possible to obtain analytical insights about the junction curvature from further approximation. By assuming that the curvature radius (1/H(s)) of the central line r(s) is almost constant, i.e., ${\left(1/H\right)}^{\prime }\ll 1$, we can write the curvature of the membranes of neighboring two cells ($H_{1}$ and $H_{2}$) as $H_{1}=H+l^{\prime \prime }/2,H_{2}=H-l^{\prime \prime }/2$. In the absence of F-actin retrograde flow, the governing equation for l(s) is then obtained from Eqs. 1 and 2:

(9) $$l^{\prime \prime }-\left(\frac{1}{f_{1}}+\frac{1}{f_{2}}\right)k\frac{l-l_{0}}{l_{0}}-\left(\frac{P_{1}}{f_{1}}+\frac{P_{2}}{f_{2}}\right)=0\text{.}$$

If we set the boundary conditions to be $l{|}_{x=0}=l{|}_{x=x_{0}}=l_{0}$, the expression for *l* (or strain ε) is

(10) $$\varepsilon =\frac{l-l_{0}}{l_{0}}=\frac{\beta_{1}}{\alpha_{1}}\left(\frac{e^{\sqrt{\alpha_{1}}\tilde{s}}}{1+e^{\sqrt{\alpha_{1}}}}+\frac{e^{-\sqrt{\alpha_{1}}\tilde{s}}}{1+e^{-\sqrt{\alpha_{1}}}}-1\right)\text{,}$$

where $\tilde{s}=s/s_{0}$ is the dimensionless arc length of central line, $\alpha_{1}=1/\tilde{f_{1}}+1/\tilde{f_{2}},\beta_{1}=\tilde{P_{1}}/\tilde{f_{1}}+\tilde{P_{2}}/\tilde{f_{2}},\alpha_{2}=1/\tilde{f_{1}}-1/\tilde{f_{2}},\beta_{2}=\tilde{P_{1}}/\tilde{f_{1}}-\tilde{P_{2}}/\tilde{f_{2}}$. These are combined parameters related to dimensionless pressure ($\tilde{P}$) and tension ($\tilde{f}$). Based on the solution for *l*, the curvature of the central line is

(11) $$H\left(\tilde{s}\right)=\frac{l_{0}}{2s_{0}^{2}}\left[\beta_{2}+\frac{\alpha_{2}\beta_{1}}{\alpha_{1}}\left(\frac{e^{\sqrt{\alpha_{1}}\tilde{s}}+e^{\sqrt{\alpha_{1}}\left(1-\tilde{s}\right)}}{1+e^{\sqrt{\alpha_{1}}}}-1\right)\right]\text{;}$$

in particular, the curvature at the junction midpoint ($H_{m}=H\left(\tilde{s}=1/2\right)$) is

(12) $$H_{m}=\frac{l_{0}}{2s_{0}^{2}}\left[\beta_{2}+\frac{\alpha_{2}\beta_{1}}{\alpha_{1}}\left(\frac{2e^{\sqrt{\alpha_{1}}/2}}{1+e^{\sqrt{\alpha_{1}}}}-1\right)\right]\text{.}$$

It is clear that when $\tilde{f_{1}}=\tilde{f_{2}},H=\frac{\left(\tilde{P_{1}}-\tilde{P_{2}}\right)l_{0}}{2\tilde{f}s_{0}^{2}}$ is constant along the junction, and the shape of curvature is completely determined by the pressure difference and surface tension. A larger pressure difference and smaller tension give larger curvature. On the other hand, when $\tilde{P_{1}}=\tilde{P_{2}}$, the curvature is spatially varying, and the midpoint curvature is $H_{m}=\frac{\tilde{P}l_{0}\alpha_{2}}{2s_{0}^{2}\left(e^{-\sqrt{\alpha_{1}}/2}+e^{\sqrt{\alpha_{1}}/2}\right)}$, which has a more complicated dependence on pressure and tension.

We can also obtain analytical solutions for the junction curvature with retrograde flow. Under the same assumption as the previous paragraph, we assume that the retrograde flow velocities in two cells are described by $v_{nn,1}\left(s\right)$ and $v_{nn,2}\left(s\right)$. From Eqs. 1 and 2, we can obtain the governing equation for E-cadherin length as

(13) $$l^{\prime \prime }-\left(\frac{1}{f_{1}}+\frac{1}{f_{2}}\right)k\frac{l-l_{0}}{l_{0}}-\left(\frac{P_{1}-u_{f}v_{nn,1}}{f_{1}}+\frac{P_{2}-u_{f}v_{nn,2}}{f_{2}}\right)=0\text{,}$$

which gives *ε* as

(14) $$\varepsilon =\left(v_{1}\left(s\right)+C_{1}\right)e^{\sqrt{\alpha_{1}}s}+\left(v_{2}\left(s\right)+C_{2}\right)e^{-\sqrt{\alpha_{1}}s}\text{,}$$

where $v_{1}\left(s\right)=\frac{1}{2\sqrt{\alpha_{1}}}\int e^{-\sqrt{\alpha_{1}}s}\beta_{3}\left(s\right)ds$ and $v_{2}\left(s\right)=-\frac{1}{2\sqrt{\alpha_{1}}}\int e^{\sqrt{\alpha_{1}}s}\beta_{3}\left(s\right)ds$. $\alpha_{1}$ is defined the same as before, and $\beta_{3}=\frac{\tilde{P_{1}}-\mu_{f}v_{nn,1}/k}{\tilde{f_{1}}}+\frac{\tilde{P_{2}}-\mu_{f}v_{nn,2}/k}{\tilde{f_{2}}}$. $C_{1}$ and $C_{2}$ are integration constants which are determined by boundary conditions (E-cadherin length at junction ends). Further, we can write the junction curvature as

(15) $$H=\frac{l_{0}}{2s_{0}^{2}}\left[\beta_{4}+\alpha_{2}\left(\left(v_{1}\left(s\right)+C_{1}\right)e^{\sqrt{\alpha_{1}}s}+\left(v_{2}\left(s\right)+C_{2}\right)e^{-\sqrt{\alpha_{1}}s}\right)\right]\text{,}$$

where $\beta_{4}=\frac{\tilde{P_{1}}-\mu_{f}v_{nn,1}/k}{\tilde{f_{1}}}-\frac{\tilde{P_{2}}-\mu_{f}v_{nn,2}/k}{\tilde{f_{2}}}$.

#### Effects of pressure and surface tension on junction curvature

**Figure 4:**
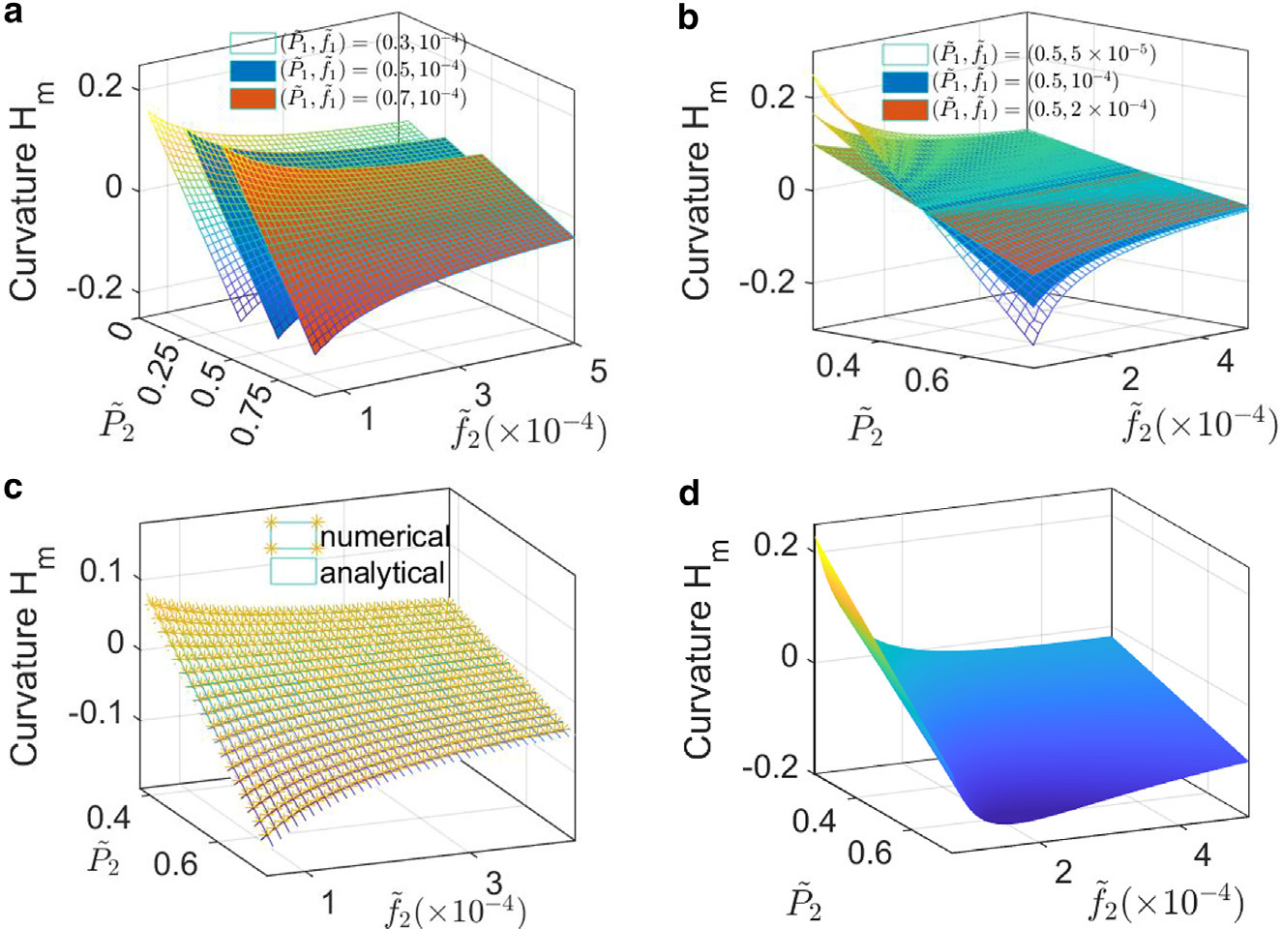
Parameter scan of how pressure, tension, and F-actin retrograde flow influence cell-cell junction curvature. (*a* and *b*) Influence of tension and pressure difference on curvature surface without actin retrograde flow. When $P_{1}$ and $f_{1}$ are fixed, larger $f_{2}$ gives smaller curvature while a larger pressure difference gives larger curvature. Pressure influences the curvature only by pressure difference, but two tensions influence the curvature separately. (*c*) Comparison between numerical result and analytical approximation. In the calculation, pressure and tension in cell 1 are fixed: $\tilde{P_{1}}=0.5,\tilde{f_{1}}=10^{-4}$. (*d*) Influence of tension and pressure on curvature with actin retrograde flow. The curvature is nonmonotonically dependent on tension $f_{2}$ due to the trade-off between tension and frictional force caused by retrograde flow. In the calculation, pressure and tension in cell 1 are fixed: $\tilde{P_{1}}=0.5,\tilde{f_{1}}=10^{-4}$. Actin polymerization rate is set as constant: $J_{1}=J_{2}=1$ (s^−1^*μ*m^−2^). To see this figure in color, go online.

To obtain an overall understanding of how different parameters influence the junction shape, we perform a parameter scan of tension ($\tilde{f_{2}}$) and pressure($\tilde{P_{2}}$) when fixing $\tilde{f_{1}},\tilde{P_{1}}$ (Fig. 4). The shape is represented by the curvature at the midpoint of the central line. Fig. 4, *a*–*c* are the results without retrograde flow, in which we change $\tilde{P_{1}}$ and $\tilde{f_{1}}$, respectively. When fixing $\tilde{f_{1}}$ and $\tilde{P_{1}},\tilde{P_{2}}$ decreases the signed curvature and this influence is enlarged with lower tension $\tilde{f_{2}}$. When fixing $\tilde{P_{2}}$, the absolute value of curvature decreases with the increase of tension $\tilde{f_{2}}$, and this effect is more obvious with larger pressure differences. In Fig. 4 *a*, the surfaces are parallel to each other when changing $\tilde{P_{1}}$, which means the pressures $\tilde{P_{1}},\tilde{P_{2}}$ do not influence the geometry separately. Instead, the pressure difference $\tilde{P_{1}}-\tilde{P_{2}}$ determines the shape. A larger pressure difference gives larger curvature. In Fig. 4 *b*, the surfaces intersect each other at the line $\tilde{P_{1}}=\tilde{P_{2}}=0.5$. This means that when pressures are the same, tension has little influence on the junction shape. When the pressures are not equal, $\tilde{f_{1}}$ and $\tilde{f_{2}}$ influence the shape independently. Fig. 4 *c* shows the comparison between numerical results and analytical approximation: The two results agree with each other, implying that the constant curvature assumption is satisfied in this parameter regime. In this calculation, E-cadherin stiffness is set as k=1.5 kPa.

Interestingly, when the retrograde flow is included, the frictional force between actin and E-cadherin can cause a nonmonotonic change in junction curvature (Fig. 4 *d*). This is due to a trade-off between tension and frictional force caused by retrograde flow. When fixing $\tilde{P_{2}}$ (let $\tilde{P_{2}}>\tilde{P_{1}}$) and increasing F-actin concentration $\theta_{n},\tilde{f_{2}}$ increases, but the retrograde flow rate $v_{nn}$ decreases according to the continuity equation $\theta_{n}v_{nn}=J_{n}$. With increasing F-actin concentration $\theta_{n}$, the decrease in retrograde flow rate ($v_{nn}$) leads to an increase in the absolute value of curvature due to a reduction in the frictional force exerted toward the cell interior. Consequently, the magnitude of the bending deformation increases. The junction bends upward due to the negative curvature. When $\theta_{n}$ is further increased, the increase in tension becomes more important, resulting in a decrease in the absolute value of curvature and, therefore, the bending deformation is reduced. Despite this decrease, the negative curvature persists, causing the junction to continue bending upward. We note that these results depend on how actin flow and F-actin concentration are related to cortical tension. Detailed models of these variables are currently not available. Pressure has the same effect as without retrograde flow. Here we assume that tension ($\tilde{f}$) is proportional to F-actin concentration ($\theta_{n}$ $\left({\mu m}^{-3}\right)$) and that the velocity of retrograde flow is inversely proportional to concentration based on the continuity equation. The polymerization rate is set as constant: $J_{1}=J_{2}=1$ (s^−1^ *μ*m^−2^), frictional coefficient $\mu_{f}=1$ (kPa · s/*μ*m). $f=\zeta \theta_{n}$, where ζ=1 (kPa ·μm^4^). Pressure and tension in cell 1 are set as $\tilde{P_{1}}=0.5$ and $\tilde{f_{1}}=0.1$ (Table 1).

**Table 1:** Glossary of model variables and values in simulation

Notation	Description	Value in simulation
$P_{i}\left(i=1,2\right)$	hydrostatic pressure in cell *i* (kPa)	0.75–1.5
$f_{i}$	combined cortical tension (kPa ·μm)	1.5–15
*k*	stiffness of E-cadherin (kPa)	1.5
$\mu_{f}$	frictional coefficient (kPa · s/*μ*m)	1
$l_{0}$	rest length of E-cadherin (*μ*m)	0.01
*h*	thickness of cortical layer (*μ*m)	1
*ζ*	surface tension coefficient (kPa ·μm^4^)	1
*α*	rate constant of water transport (*μ*m/(kPa · s))	$10^{4}–10^{5}$
*β*	rate constant of passive ion transport (m/s)	$5\times 10^{5}$
*γ*	rate constant of active transport (mol/(${\mu m}^{2}$ s · kPa))	$5\times 10^{-4}$

See text for references.

### Actin tangential flow and a dynamical model of cell-cell junction movement

In the previous section, we only considered the effects of retrograde flow perpendicular to the cell membrane. However, in a live cell, there is E-cadherin movement along the membrane, which is coupled to the tangential movement of F-actin due to attachment between actin and cadherin through *α*- and β-catenin and other molecules. E-cadherin molecules also interact with each other, including both *cis* and *trans* interactions, which work cooperatively (35,36) to generate E-cadherin clustering and other collective effects. The cooperative aspect of E-cadherin dynamics has been demonstrated extensively both experimentally and theoretically (37,38,39,40). Here, we neglect cooperative aspects of E-cadherin bonds (*cis* and *trans* interactions) and only consider the convection movement of E-cadherin coupled with F-actin flow. This results in tangential movements of F-actin and E-cadherin, together with spatial variations of membrane tension caused by the tangential frictional force exerted by actin network. The cortical tension is actively controlled by the cell (41). In addition to force balance in the normal direction, there is also tangential force balance:

(16) $$\left(P_{1}-\mu_{f}v_{nn,1}\right)-\frac{\left(T_{1}+\sigma_{1}h\right)y_{1}^{\prime \prime }}{\sqrt{\left(1-{y^{\prime }}_{1}^{2}\right)}}+\frac{k}{l_{0}}\left(\sqrt{{\left(x_{1}-x_{2}\right)}^{2}+{\left(y_{1}-y_{2}\right)}^{2}}-l_{0}\right)=0\text{,}$$

(17) $$\left(P_{2}-\mu_{f}v_{nn,2}\right)+\frac{\left(T_{2}+\sigma_{2}h\right)y_{2}^{\prime \prime }}{\sqrt{\left(1-y_{2}^{\prime 2}\right)}}+\frac{k}{l_{0}}\left(\sqrt{{\left(x_{1}-x_{2}\right)}^{2}+{\left(y_{1}-y_{2}\right)}^{2}}-l_{0}\right)=0\text{,}$$

(18) $$x_{1}^{\prime }=\sqrt{1-{y^{\prime }}_{1}^{2},}$$

(19) $$x_{2}^{\prime }=\sqrt{1-{y^{\prime }}_{2}^{2},}$$

(20) $$T_{1}^{\prime }=-\mu_{f}v_{nt,1}\text{,}$$

(21) $$T_{2}^{\prime }=-\mu_{f}v_{nt,2}$$

where $v_{nn}$ and $v_{nt}$ are the normal and tangential components of actin velocity at the cell membrane. The boundary conditions for these equations are the same as before (fixed E-cadherin length at both ends), except that there are additional conditions for membrane tension: $T_{1}\left(0\right)=T_{1b}$ and $T_{2}\left(0\right)=T_{2b}$. Since actin-bound E-cadherin is anchored into the actin network, the velocity of bound E-cadherin equals actin tangential velocity $v_{nt}$. The lateral diffusion of E-cadherin is constrained (42,43), so here we neglect the E-cadherin diffusion and only consider the convection; therefore, the movement can simply be described as

(22) $$\frac{\partial \rho_{E}\left(s,t\right)}{\partial t}=-\frac{\partial }{\partial s}\left(v_{nt}\rho_{E}\right)\text{,}$$

where $\rho_{E}$ is the E-cadherin bond density, and E-cadherin velocity $v_{nt}$ is the average of tangential actin velocities in two cells at the membrane: $v_{nt}=\left(v_{nt,1}+v_{nt,2}\right)/2$. To close the equation, E-cadherin number conservation is assumed: $\int_{0}^{s_{0}}\rho_{E}ds=C$, in which *C* is a constant. It is also possible to introduce binding kinetics of E-cadherin with F-actin, a dynamic population of bound E-cadherins, and cooperative binding between E-cadherin bonds. For example, if E-cadherin bonds can dynamically form and break under force, then the population of E-cadherin bonds is not conserved, and kinetic rate terms can be introduced in Eq. 22. E-cadherin bonds are known to be catch bonds (20,44), which can be modeled by bond dissociation rates that are declining functions of bond tension. There is also feedback between actomyosin contractility and cadherin-based adhesions (45,46,47), where the E-cadherin density might change in response to the actin retrograde flow rate. When E-cadherin bond density can increase and decrease, the junction length can also change. This would require a moving boundary treatment of the junction region where the domain of *s* can change. These aspects will further complicate the model and add additional parameters.

**Figure 5:**
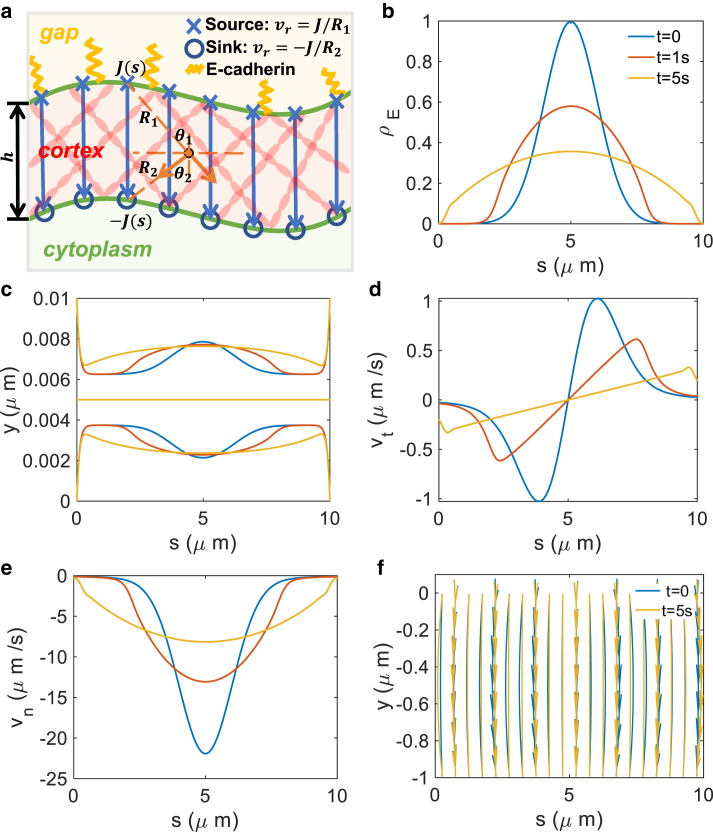
Dynamical results of junction mediated by actin flow and E-cadherin movement. (*a*) Schematic illustration of the junction structure and potential flow assumption. (*b*) Evolution of E-cadherin density distribution. (*c*) Evolution of cell membrane and junction shape (*central line*). There is wave propagation from the middle to both ends. (*d* and *e*) Evolution of tangential and normal velocity profile. (*f*) Change of streamlines at t=0 and t=5s. In the calculation, the initial E-cadherin bond distribution is Gaussian: $\rho_{E}\left(s,0\right)=1/\sqrt{2\pi }\times e^{-{\left(s-5\right)}^{2}/2}$. E-cadherin number is assumed to be conserved at all times. The simulation time is 5 s. E-cadherin stiffness is set as $k=1+0.5\rho_{E}$, the cortical tension is a constant: σh=1.5 (kPa ·μm), the membrane tension at the left end is T(0)=0.5 (kPa ·μm). The polymerization rate is $J=\rho_{E}$, and the frictional coefficient is set as μ=0.005 (kPa · s/*μ*m). Parameters for the two cells are the same (see Table 1). To see this figure in color, go online.

In general, there are both normal and tangential components for the actin velocity in the cortex. Here, we have assumed that the F-actin network is incompressible. Moreover, retrograde flow is mostly the result of actin polymerization at the membrane and depolymerization at the inner edge of the cortex. There is very little rotational motion in the flow. Therefore, we can use the potential flow approximation in the presence of sources and sinks (48). For a single source/sink of strength *J*, the flow is in the radial direction. In two dimensions the radial velocity is J/R, where *R* is the distance from the source/sink. To satisfy mass balance, the strength of the source should be the same as that of the sink: $J_{source}\left(s\right)=J_{sink}\left(s\right)$. The velocity field inside the cortex can then be expressed as

(23) $$v_{x}\left(x,y\right)=\int_{0}^{s_{0}}\left(\frac{J\left(s\right)}{R_{1}}\sin\;\theta_{1}-\frac{J\left(s\right)}{R_{2}}\sin\;\theta_{2}\right)ds\text{,}$$

(24) $$v_{y}\left(x,y\right)=\int_{0}^{s_{0}}\left(\frac{J\left(s\right)}{R_{1}}\cos\;\theta_{1}+\frac{J\left(s\right)}{R_{2}}\cos\;\theta_{2}\right)ds\text{,}$$

where $R_{i}$ and $\theta_{i}$ are geometrical quantities shown in Fig. 5 *a*. In general, E-cadherin bonds can be partitioned into unbound and actin-bound portions. The portion connected to the actin network moves with actin flow, while the unbound portion is not anchored into the actin network and can freely diffuse. The density $\rho_{E}$ describes the actin-bound E-cadherin bonds. The actin-bound E-cadherin bonds can also nucleate new actin filaments (18,49); therefore, the polymerization rate is $J\left(s\right)=\eta_{1}\rho_{E}\left(s\right)$. E-cadherin bond stiffness, *k*, should be proportional to total E-cadherin density: $k\left(s\right)=\eta_{2}\rho_{E}\left(s\right)+\eta_{3}$. $\eta_{3}$ accounts for the background stiffness from unbound E-cadherins. The coupled equations above can be solved numerically. The numerical solution requires two steps. First, we initialize the E-cadherin density distribution $\rho_{E}$, from which we compute the actin velocity field in the cortex and the shape of two membranes. Second, we update the E-cadherin density distribution $\rho_{E}$ based on Eq. 22. These two steps are repeated to obtain the time evolution of cell-cell junction shapes and E-cadherin distribution.

We can simulate the time evolution of the cell-cell junction as actin-bound E-cadherin is redistributed. We assume quasi-static equilibrium, i.e., the force balance is satisfied at all times and there is no fluid flow around the cell-cell junction. This assumption is relaxed in the next section where we consider time evolution due to fluid motion around the junction. We start with a Gaussian-distributed E-cadherin density: $\rho_{E}\left(s,0\right)=1/\sqrt{2\pi }\times e^{-{\left(s-5\right)}^{2}/2}$ (Fig. 5 *b*). Our choice of the initial distribution is motivated by the assembly process of adherens junctions, which starts with the binding of E-cadherin at the contact points between cells, followed by the spreading of E-cadherin bonds from the middle to both ends of the junction (50). Although there is no direct experimental evidence for this exact form of the initial distribution, we use the Gaussian distribution to approximate the initial density at the contact point. As time progresses, the density distribution becomes wider because the actin velocity tangent to the junction points outward from the center to the edge (Fig. 5, *b* and *d*). This is because the E-cadherin bond density and the source (polymerization) strength are higher at the center than at the ends. After a long time, the cadherin density becomes evenly distributed. Accompanied by the E-cadherin spreading process, there is a propagation of membrane curvature from the center to both ends, as is shown in Fig. 5 *c*. However, due to symmetry, the centerline or the junction shape remains unchanged. Fig. 5, *d* and *e* show the tangential ($v_{t}$) and normal ($v_{n}$) components of the actin velocity at the cell membrane of cell 2 (the lower cell). Similar to E-cadherin density distribution, the retrograde flow rate (normal velocity) has a wider distribution as time progresses. The tangential flow profile exhibits two peaks propagating outward from the center to both ends, and the magnitude decreases with time as the E-cadherin becomes more evenly distributed. By the end of the simulation at t=5 s, the velocity profile is almost linear, with small discontinuity at the edges. Since the velocity field is calculated by superposition of velocity field contributed by each point on the cell membrane, the overall velocity field is dependent on the geometry of cell membranes. Since the curvature changes significantly near the edges, the slight discontinuity of tangential velocity field is reasonable. Fig. 5 *f* shows the streamline of the actin network inside the cortex of cell 2. Corresponding to tangential velocity distribution, the streamline becomes narrower in the middle and slightly wider at both ends. In the calculation the simulation time is 5 s, which is on the same order of magnitude as the relevant biological processes: e.g., E-cadherin binding (51), overall turnover (52), and actin flow (53). E-cadherin stiffness is set as $k=1+0.5\rho_{E}$, the cortical tension is a constant: σh=1.5 (kPa ·μm), and the membrane tension at the left end is T(0)=0.5 (kPa ·μm). The polymerization rate is $J=\rho_{E}$ and the frictional coefficient is set as μ=0.005 (kPa · s/*μ*m). Parameters for the two cells are the same (see Table 1). These results show that actin polymerization coupled to the E-cadherin distribution can influence the cell-cell junction shape and dynamics. Notably, in this simulation the timescale is 5 s for junction dynamics involving tangential actin flow, which is less than the timescale of cortex turnover. Therefore, the bending rigidity does contribute to the junction shape. For simplicity, we neglect this effect in the calculation.

### Dynamics of cell-cell junction movement: Stokeslet and immersed boundary approach

**Figure 6:**
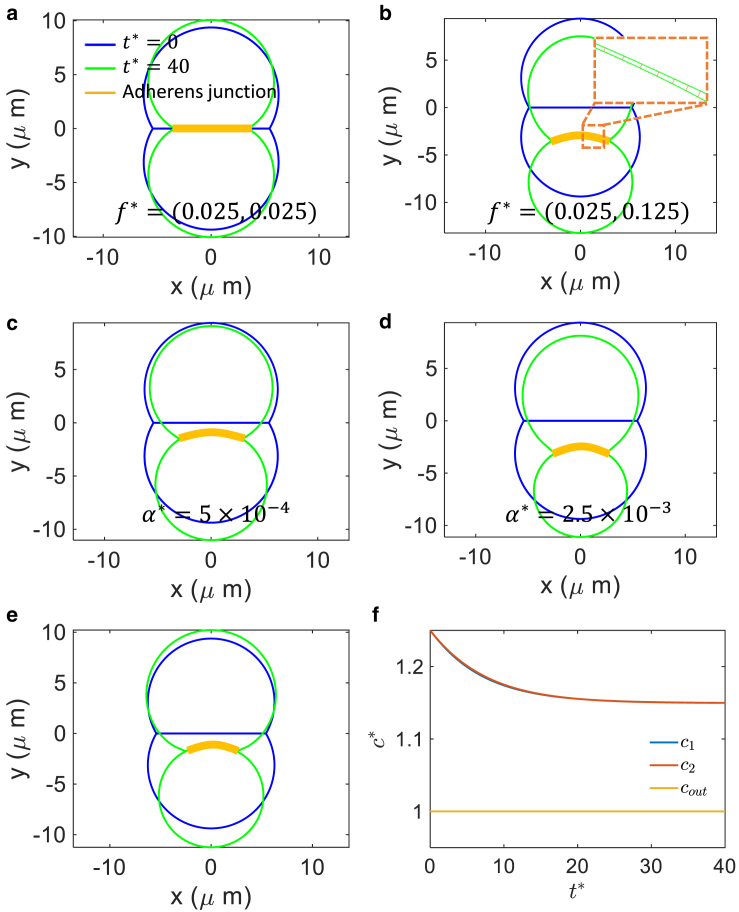
Initial and equilibrium configuration of the cell doublet under different situations. (*a* and *b*) Results of impermeable cells with equal and unequal tension: $f_{1}^{*}=f_{2}^{*}=0.025$ in (*a*) and $\left(f_{1}^{*},f_{2}^{*}\right)=\left(0.025,0.125\right)$ in (*b*). The zoom-in picture shows the junction structure near the right end. (*c* and *d*) Results of permeable cells with no ion flux and with different permeability constants. Parameters are set as $\left(f_{1}^{*},f_{2}^{*}\right)=\left(0.025,0.125\right)$ and only membrane permeability varies: $\alpha^{*}=5\times 10^{-4}$ in (*c*) and $2.5\times 10^{-3}$ in (*d*). (*e*) Results for permeable cells with both passive and active ion flux. (*f*) Evolution of ion concentration inside and outside the cells. The tension is set as $\left(f_{1}^{*},f_{2}^{*}\right)=\left(0.025,0.125\right)$. The permeability constant and the ion transport coefficients are set as $\alpha^{*}=2.5\times 10^{-3},\gamma^{*}=1.25\times 10^{-9},\beta^{*}=1.25\times 10^{-3}$. To see this figure in color, go online.

In previous sections, we explored a quasi-static mechanical equilibrium model of the adherens junction. No forces (except hydrostatic pressure) from the surrounding fluid are considered. However, cells are always immersed in a fluid environment. Because the Reynolds number is very small, the contribution of fluid motion to cell motion is important. Moreover, the cell membrane is permeable to water and ions, and on the timescale of cell motion water and ion flows across the cell membrane are important driving forces for cell boundary motion (54,55). In this section, we focus on a cell doublet forming a junction at the contact region (Fig. 6). We treat the two cells as enclosed permeable surfaces immersed in a fluid. Cells can exert active tension and influence both the fluid motion and the junction shape. Same as before, the E-cadherin bonds are modeled as elastic springs, the force of which is proportional to the bond strain. The E-cadherin bonds connect the two adjacent cells via fixed discretized points along the cell-cell junction.

For an immersed and water-permeable interface in a viscous fluid, the interface moves with the fluid flow according to the equation (56):

(25) $$\frac{dX}{dt}=u-J_{w}\text{,}$$

where X is the Lagrangian coordinate of the membrane points, u is the fluid velocity, and $J_{w}$ is the water flux across the cell membrane. $J_{w}$ is normal to the membrane surface and is determined by both hydrostatic pressure difference Δp and osmotic pressure difference ΔΠ across the membrane: $\left|J_{w}\right|=\alpha \left(\Delta p-\Delta \Pi \right)$ (56), where *α* is the cell-membrane permeability. Specifically, in the junction region, we only consider direct water and ion transport between the two cells and do not consider water and ion dynamics within the gap. For the simplest case, fluid movement around the cell junction can be treated using Stokes flow:

(26) $$-\nabla p\left(x\right)+\mu \Delta u\left(x\right)=-F_{mf}\text{,}$$

(27) ∇·u=0,

where *p* is the hydrostatic pressure, *μ* is fluid viscosity, and $F_{mf}$ is the body force exerted by the cell membrane onto the fluid, which is determined by

(28) $$F_{mf}=\int_{\Gamma }\left[f\frac{d^{2}X}{ds^{2}}+\frac{k}{l_{0}}\left(X_{p}-X-l_{0}\frac{X_{p}-X}{\left|X_{p}-X\right|}\right)\right]\delta \left(x-X\right)ds\text{,}$$

where *f* is the surface tension, which is actively controlled and is treated as constant for the same cell. *k* is the stiffness of the E-cadherin bond and $X_{p}$ is the point connected via E-cadherin bond. For points without E-cadherin bonds, $X_{p}=X$. δ(x) is the Dirac function, and the integral means the interpolation from membrane force to fluid body force.

The osmotic pressure is mainly determined by solute/ion concentrations in the medium. For simplicity, we assume there is only one type of solute and that solute diffusion is rapid, given an evenly distributed solute concentration inside $\left(c_{1},c_{2}\right)$ and outside $\left(c_{out}\right)$ of the cells. The solute dynamics can be written as a set of ordinary differential equations:

(29) $$\frac{d\left(c_{i}A_{i}\right)}{dt}=\int_{0}^{L_{i}}\left(J_{a,i}+J_{p,i}\right)ds\;\left(i=1,2\right)\text{,}$$

where $A_{i}$ is the cell area and $J_{a},J_{p}$ are ion flux induced by active pumps and passive channels. Details of cell pump and leak dynamics are complex and have been discussed elsewhere (57,58,59). For this paper, for illustration purposes we use the phenomenological model introduced in (60), where $J_{p,i}=\beta \left(c_{out}-c_{i}\right)$ and $J_{a,i}=\gamma \left({\Delta \Pi }_{c}-\Delta \Pi \right)$. $\Delta \Pi =\Pi_{in}-\Pi_{out}$ is the osmotic pressure difference between the inside and outside of the cell doublet. ${\Delta \Pi }_{c}$ is a critical osmotic pressure difference given by zero free-energy change (ΔG), which is approximately ${\Delta \Pi }_{c}=6\times 10^{4}\Pi_{out}$ (60). In the junction region, we assume there is only direct ion transport between two cells and there is only passive transport whose flux is proportional to $c_{2}-c_{1}$. $L_{i}$ is the perimeter of cell *i*.

The coupled fluid flow and membrane movement described by Eqs. 25, 26, and 27 is a moving boundary problem, for which many numerical methods have been developed (61,62,63). Under the quasi-static assumption that both the velocity field and cell shape change slowly, the membrane shape and velocity field can be updated iteratively. In our case, we utilize the regularized Stokes method (64) to solve for the pressure and fluid velocity fields. This method is grid-free and easy to implement, and numerically efficient. Specifically, the Stokeslet method solves the flow field driven by external body force. The steady-state Stokes equations are described by Eqs. 26 and 27. The fundamental solution (Green’s function) to these equations is the Stokeslet, which describes the fluid field caused by a singular point force: $F_{mf}=F\delta \left(r-r_{0}\right)$. The velocity and pressure are assumed to vanish at infinity. Owing to the linearity of Stokes equations, the flow pattern caused by any body force distribution can be obtained via a superposition of the fundamental solutions. In two dimensions, the pressure and velocity Stokeslets are

(30) $$p\left(r\right)=\frac{F\cdot \left(r-r_{0}\right)}{2\pi {\left(r-r_{0}\right)}^{2}}\text{,}$$

(31) $$u\left(r\right)=-\frac{F}{4\pi \mu }\ln\left(\frac{\left|r-r_{0}\right|}{L}\right)+\left[F\cdot \left(r-r_{0}\right)\right]\frac{r-r_{0}}{4\pi \mu {\left(r-r_{0}\right)}^{2}}\text{,}$$

where *L* is a scale factor, which is set to be L=20μm, or the typical cell size. Note that there are singularities in the 2D pressure and velocity fields at the location of body force. The singularity makes it numerically difficult to obtain the velocity field near the body force. In our case, to update the cell position, we have to obtain the fluid velocity at the membrane, which is also the location of the body force. One technique for dealing with this problem is to introduce a regularized kernel for point force that includes a small cutoff parameter *ε*. In this approach, the point force is spread out over a small ball. The size of the ball is described by the parameter *ε*. In two dimensions, a typical choice of the spread function is $\phi_{\varepsilon }\left(r\right)=\frac{3\varepsilon^{3}}{2\pi {\left({\left|r\right|}^{2}+\varepsilon^{2}\right)}^{5/2}}$. The point force is then replaced by $F\phi_{\varepsilon }\left(r-r_{0}\right)$. All the numerical details can be found in (64).

It is convenient to nondimensionalize all quantities in equations in this section, with dimensionless parameters to be $x^{*}=x/L,p^{*}=p/P,u^{*}=\frac{\mu }{PL}u,t^{*}=\frac{P}{\mu }t,f^{*}=\frac{f}{PL},k^{*}=k/P,\alpha^{*}=\frac{\mu \alpha }{L},\beta^{*}=\frac{\mu \beta }{PL^{2}},\gamma^{*}=\frac{\mu \gamma }{CL^{2}}$, where L,P,C are scale constants set as L=20 *μ*m, P=1 kPa, and *C* = 1 mM. Using the regularized Stokeslet approach and Eqs. 25, 26, 27, 28, and 29, we compute the cell-cell junction shape dynamics over time. Fig. 6 shows the initial and the final static equilibrium configuration of the cell doublet in different situations. Fig. 6, *a* and *b* are results without water permeability: i.e., $\alpha^{*}=0$. Different tension parameters $f^{*}$ are applied. With equal tension $f_{1}^{*}=f_{2}^{*}=0.025$, the junction is flat and only has contraction in the initial straight line. When a tension difference is introduced, $\left(f_{1}^{*},f_{2}^{*}\right)=\left(0.025,0.125\right)$, the junction bends toward the cell with lower tension due to lower hydrostatic pressure. Interestingly, there is an overall downward movement of the cell doublet. Owing to impermeability, the cells maintain the same areas. However, when membrane water permeability is included but ionic fluxes are not included, both cells shrink in size because the inner pressure drives water out. Fig. 6, *c* and *d* show results with membrane water permeability and tension difference ($\alpha^{*}=5\times 10^{-4},2.5\times 10^{-3}$ and $\left(f_{1}^{*},f_{2}^{*}\right)=\left(0.025,0.125\right)$). When there is a tension difference, the cell with larger tension shrinks more because it drives water flux into the other cell. Fig. 6 *e* is the result when there is passive and active ion transport, where $\alpha^{*}=2.5\times 10^{-3},\gamma^{*}=1.25\times 10^{-9}$. As expected, active transport can help to maintain cell size. Fig. 6 *f* shows the ion concentration change inside and outside the cells. In all calculations, E-cadherin stiffness is set as $k^{*}=1.5$, and ion transport coefficient is set as $\beta^{*}=1.25\times 10^{-3}$ (see Table 1). In all calculations, the dimensionless final time is $t^{*}=40$. The real timescale depends on both the reference pressure *P* and viscosity *μ*. It is important to note that the viscosity *μ* here should be interpreted as an effective parameter that also incorporates the influence of the substrate and adhesions.

**Figure 7:**
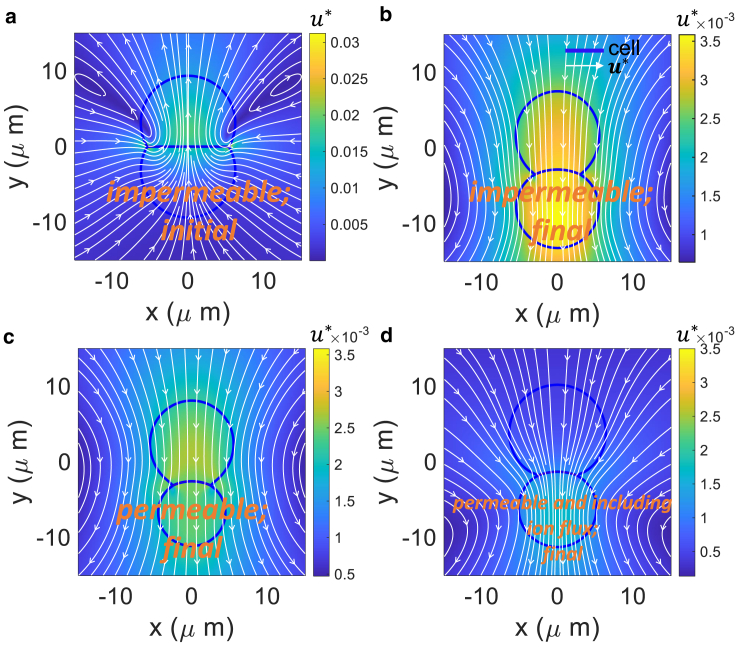
Fluid velocity fields for varying cell permeability and tension distribution. The color plot is a representation of magnitude of the dimensionless flow velocity. (*a*) Impermeable cells with different tensions when $t^{*}=0$. (*b*) Impermeable cells (final: $t^{*}=40$). In both (*a*) and (*b*), tensions are set as $\left(f_{1}^{*},f_{2}^{*}\right)=\left(0.025,0.125\right)$. (*c*) Permeable cells without ions fluxes between cells (final: $t^{*}=40$). Tension and permeability constants are set as $\left(f_{1}^{*},f_{2}^{*}\right)=\left(0.025,0.125\right),\alpha^{*}=2.5\times 10^{-3}$. (*d*) Permeable cells with both passive and active ion flux (final: $t^{*}=40$). Parameters are set as $\left(f_{1}^{*},f_{2}^{*}\right)=\left(0.025,0.125\right),\alpha^{*}=2.5\times 10^{-3},\gamma^{*}=1.25\times 10^{-9},\beta^{*}=1.25\times 10^{-3}$. To see this figure in color, go online.

A consistent prediction is that the fluid flow patterns around the cell doublet can vary dramatically depending on parameters. Initially, there are two symmetrical vortices in the upper cell (with lower tension) and the cell doublet starts deforming (Fig. 7 *a*). The junction is initially flat and the fluid velocity field is upward overall. When there is no active ion transport and time is long enough, there is a uniform flow pattern, and the cell doublet moves downward (Fig. 7, *b* and *c*). When there is active ion transport the cell doublet still has an overall downward movement, but the fluid velocity is much higher in the lower cell with larger tension ($\left|u^{*}\right|\approx 2\times 10^{-3}$ in the lower cell compared to $\left|u^{*}\right|\approx 5\times 10^{-4}$ in the upper cell.) Also, the velocity field is not uniform in the lower cell (Fig. 7 *d*). We note that these flow fields are from 2D Stokeslet calculations. 3D results will differ substantially and are potentially more realistic. The flow fields are also long-range. The 3D situation can potentially include adhesions with the substrate, extracellular matrix (ECM), and complex cell geometries.

It is interesting to examine the mechanism of cell movement driven by active flow patterns predicted by the immersed boundary approach. In general, geometrical asymmetry and tension difference will result in nonzero total force on the fluid, i.e., $\int d{rF}_{mf}\neq 0$. This excess force is balanced by friction and pressure from fluid flow around the object. The corresponding asymmetrical body force exerted on the fluid will generate flow patterns, and the object will move. Cells potentially can utilize this principle to move, either singly or in multicellular contexts. Here the downward flow pattern is due to unbalanced cortical tension in the cell doublet (which also points downward), which then is balanced by flow around the cell that pushes the cells downward. Since the junction bends toward the cell with lower tension, the total force of the junction part on the fluid points downward, which breaks the symmetry and results in the downward flow pattern. In contrast, when the junction is flat at the initial state, the cell doublet is geometrically symmetric and the total force points upward, resulting in an initial upward flow pattern. Therefore, if cells can actively control cortical tension, fluid flow is generated and movement is created. This mechanism of cell movement is purely dissipative and cannot be derived from energy minimization. Models such as the vertex model minimize an energy, which gives zero total force. Therefore the mechanism of movement here is outside of energy-based models such as the vertex model.

**Figure 8:**
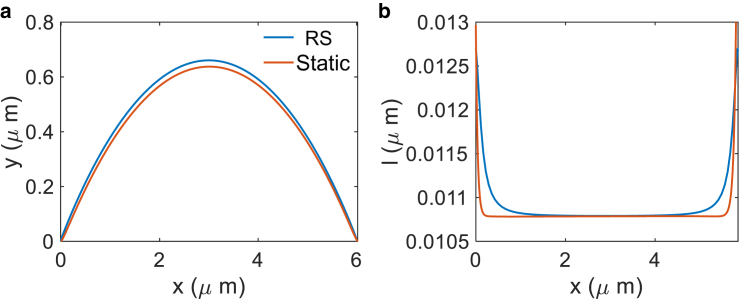
Comparison between methods of regularized Stokeslet (RS) and the static membrane mechanics model of Eqs. 1 and 2. (*a*) The computed junction shape. (*b*) E-cadherin bond length distribution. No actin dynamics is considered here. The parameters used are the same for these two methods: $\left(f_{1},f_{2}\right)=\left(0.5,2.5\right)$ kPa ·μm, $\left(P_{1},P_{2}\right)=\left(0.0732,0.4834\right)$ kPa, and k=1.5 kPa. To see this figure in color, go online.

The cell doublet with an impermeable membrane can be compared to the previous result with only membrane mechanics (Fig. 8). Both junction shape and E-cadherin length distribution are compared. With the same parameters $\left(f_{1},f_{2}\right)=\left(0.5,2.5\right)$ kPa ·μm, $\left(P_{1},P_{2}\right)=\left(0.0732,0.4834\right)$ kPa, and k=1.5 kPa, the two model results agree well with each other. Note that the pressure field is not completely uniform inside the cell in the immersed boundary model, whereas in the membrane mechanics model the pressure is assumed to be uniform. Therefore, a slight difference in E-cadherin bond length between the two models is expected.

## Discussion

In this work, we explored mechanical models of cell-cell adherens junction dynamics. We first explored a model covering membrane mechanics, actin dynamics, and E-cadherin bond mechanics. In this model, we describe E-cadherin as elastic springs mediating cell-cell contact. We also assume that the cortical tension is actively controlled by the cell and is given. The membrane tension could vary spatially due to tangential actin flow. The hydrostatic pressure is assumed to be a constant inside the cell. The result shows that dimensionless pressure ($\tilde{P}=P/k$) and cortical tension ($\tilde{f}=f/\left(ks_{0}\right)$) are the most important parameters controlling the junction shape. The cytoplasmic pressure can influence the junction shape, but only the pressure difference matters. The cortical tensions in the two cells can influence the junction shape independently. A larger pressure difference gives larger junction curvature while larger tension decreases curvature, which agrees with physical intuition. E-cadherin bonds are compressed in most junction regions without actin retrograde flow and are largely influenced by the E-cadherin stiffness *k*. On the other hand, E-cadherin stiffness has little influence on the junction shape. When the actin retrograde flow is included, we see that cortical tension has a nonmonotonic influence on junction curvature due to a trade-off between tension and frictional force from the retrograde flow. Also, because retrograde force can exert a force on E-cadherin, the E-cadherin bonds could change from a compressing state to a stretched state. Analytic approximation of junction geometry is also possible, which in the small curvature limit agrees with the numerical result.

We also explored theoretically the influence of actin tangential flow on E-cadherin junction dynamics. Here the membrane tension can vary spatially. Actin-bound E-cadherins are convected by actin tangential flow. Starting with a concentrated E-cadherin density distribution, we observe an evolution of cadherin bond distribution toward the steady state. When time is long enough, the junction reaches a steady state where the junction line is straightened and the flow pattern reverts to the typical retrograde flow pattern. These results show that the distribution of E-cadherin bonds can be coupled to actin flow. As the actin flow pattern is altered, the junction shape can change as well.

For the dynamic cell-cell junction, we can describe the cell doublet as two immersed objects which are permeable to water and allow ion transport across the membrane. The cell doublet is assumed to be in a quasi-static state. The moving fluid field is then solved by the regularized Stokeslet method. Three different membrane conditions are studied: impermeable, permeable to water but without ion transport, and permeable to water and with ion transport. We observe that when a tension difference exists between two cells, the cell junction bends to the lower tension side and there is a translational motion of the cell doublet toward the higher tension side. This result shows that when the total internal force of a moving boundary does not sum to zero, the excess force can lead to fluid flow and overall motion. Note that here we are treating the fluid around the moving boundary as Stokes flow with a single viscosity. In general, inside the cell there are at least two moving fluid phases: the cytosol and the cytoskeletal network. The cytoskeleton can polymerize and depolymerize, and flow, while water can also flow from the outside into the cytosol (54). This two-phase fluid model cannot be described by the Stokeslet. Nevertheless, the immersed boundary method suggests that when fluid is permeable across the membrane, additional dynamics related to cell motion are expected. While the results presented in this study focus on cell doublets, it is feasible to extend this methodology to multicellular cases. From a modeling perspective, the principles underlying epithelial tissue and multicellular clusters are similar to those of the two-cell doublet case. However, the computational effort required for multicellular simulations is considerably greater. This is primarily due to the larger number of cell boundary points and the increased complexity of the configurations involved. The results presented in this study are obtained from a 2D Stokeslet calculation. However, 3D results will be more realistic, based on the fact that adhesions with the substrate, ECM, and complex cell geometries can be incorporated. Additionally, the fluid velocity scaling in two dimensions follows a logarithmic relationship with distance (ln(r)), whereas in three dimensions it scales with the inverse of the distance (1/r), which tends to zero at infinity and aligns with our intuition of how flow and pressure fields behave at long range.

By incorporating fluid motion and cortical tension in a single unified modeling framework, the computed cell shape and motion can be substantially different from other modeling approaches. For example, the vertex model is an energy-based approach that uses energy minima to predict epithelial shape and mechanics. In reality, this is a simplification that ignores the fluid phase. For objects with a low Reynolds number, the movement of the object must follow the fluid velocity, and viscous effects and the fluid velocity field have an important influence on the movement and mechanics. Recently, it has been becoming clear that fluid movement has a major influence in epithelial monolayers (65). Therefore, our modeling approach provides a more realistic description of motions in multicellular situations. The model can be used to describe motion in multicellular clusters or epithelia where the observed movement is likely influenced by fluid movement in and around the cell.

The different modeling approaches in this paper for describing the cell-cell junction are limits of a more complete mechanical model wherein actin dynamics and water dynamics are coupled with E-cadherin bond movement and membrane deformation. Therefore a multiphase description of the cytoplasm and extracellular space, coupled with membrane movement and ion/solute flux across the membrane, is a general approach to describe single cells, cell-cell junctions, and multicellular clusters. The complication is that the multiphase models are nonlinear and currently there are no numerical methods that are robust for all situations. For the cell-cell junction, there is an additional multiscale problem whereby the E-cadherin bonds are small (10 nm) and the cell-membrane deformations are large. Moreover, E-cadherin molecules themselves are also molecularly complex and can undergo conformational transitions. Therefore, computationally there are additional challenges for understanding cell-cell junction mechanics. Continuum models explored in this paper show that F-actin dynamics can impact E-cadherin bond mechanics, junction shape, and cell shapes in general. Parameters of the continuum models can be linked to molecular-scale models of E-cadherin (66,67) and F-actin (68,69,70,71). These models can be assembled to develop a multiscale description of the cell-cell junction in physiologically relevant settings.

## Author contributions

Y.W. and S.X.S designed the research. Y.W. and S.X.S performed the research. Y.W. and S.X.S wrote the paper.
